# Three is a crowd

**DOI:** 10.1007/s12471-014-0585-1

**Published:** 2014-08-29

**Authors:** N. Lahrouchi, E. F. D. Wever, J. C. Balt

**Affiliations:** 1 Department of Experimental Cardiology, Academic Medical Center, University of Amsterdam, Meibergdreef 15, Room L2-109, 1105 AZ Amsterdam, the Netherlands; 2 Department of Cardiology, St Antonius Hospital, PO Box 2500, 3430 EM Nieuwegein, the Netherlands

## Question

**Fig. 1:**
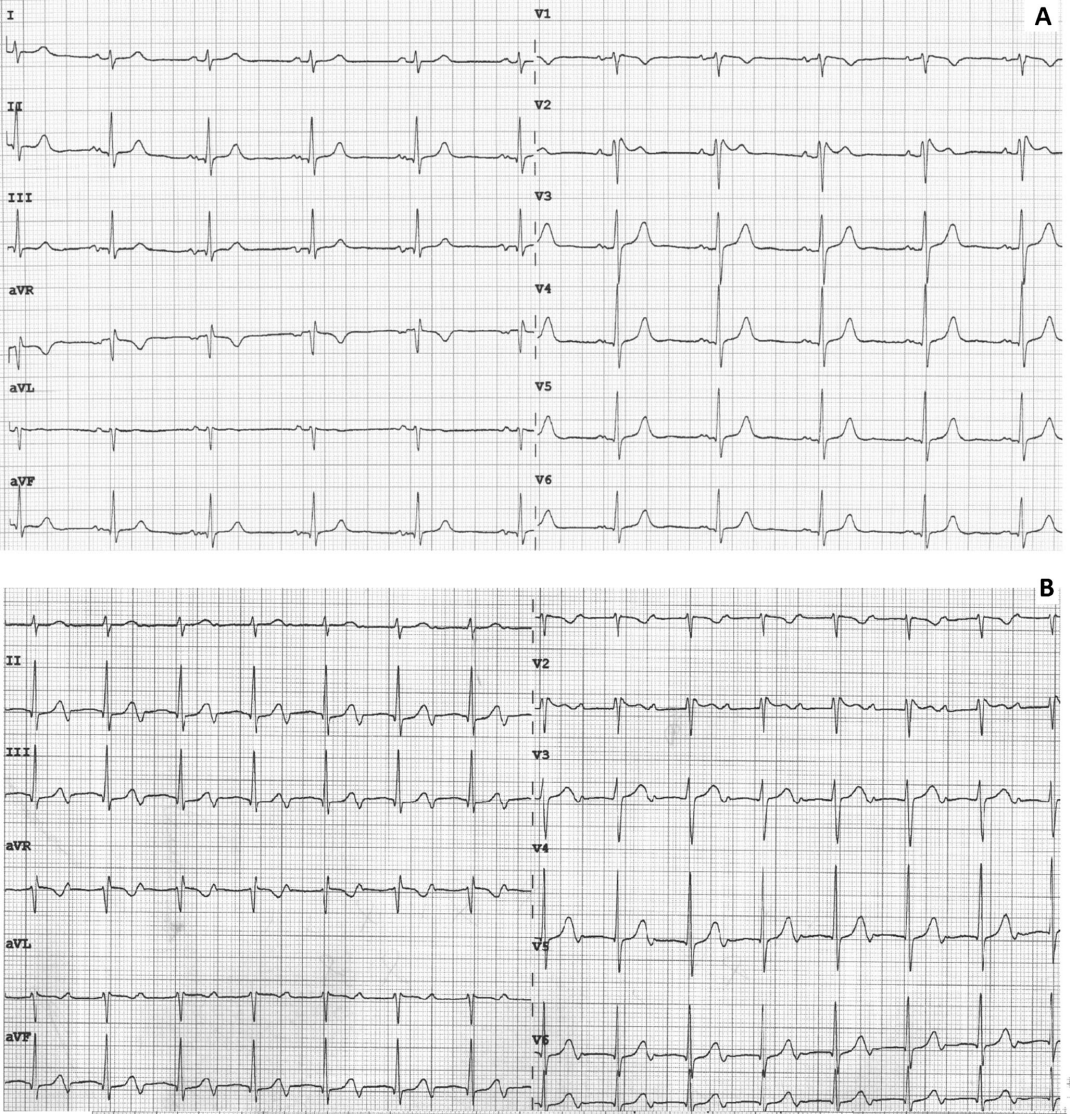
**a** ECG at baseline. **b** ECG during symptoms

A 40-year-old woman was seen at our outpatient clinic because of episodes of palpitations that lasted several hours. She reported no syncopal episodes or family history of sudden cardiac death and was not on any medication. The resting ECG and ECG recorded during the palpitations are shown in Fig. 1a and b. What is the (differential) diagnosis?

## Answer

You will find the answer elsewhere in this issue.

